# Integrated care pathway for rectal cancer treatment: cross-sectional post-implementation study using a logic model framework

**DOI:** 10.1590/1516-3180.2018.0364160919

**Published:** 2020-01-13

**Authors:** Silvia Takanohashi Kobayashi, Alessandro Gonçalves Campolina, Maria del Pilar Estevez Diz, Patrícia Coelho de Soárez

**Affiliations:** I MD, MSc. Ophthalmologist, Centro de Investigação Translacional em Oncologia, Instituto do Câncer do Estado de São Paulo (ICESP), Faculdade de Medicina FMUSP, Universidade de Sao Paulo, Sao Paulo, SP, BR.; II MD, MSc, PhD. Scientific Researcher, Centro de Investigação Translacional em Oncologia, Instituto do Câncer do Estado de São Paulo (ICESP), Faculdade de Medicina FMUSP, Universidade de Sao Paulo (FMUSP), Sao Paulo, SP, BR.; III MD, PhD. Attending Physician, Centro de Investigação Translacional em Oncologia, Instituto do Câncer do Estado de São Paulo (ICESP), Faculdade de Medicina FMUSP, Universidade de Sao Paulo, Sao Paulo, SP, BR.; IV MPH, PhD. Associate Professor, Department of Preventive Medicine, Faculdade de Medicina FMUSP, Universidade de Sao Paulo, Sao Paulo, SP, BR.

**Keywords:** Rectal neoplasms, Interdisciplinary studies, Disease management, Implementation science, Decision making, Qualitative research, Line of care, Chronic non-communicable diseases, Healthcare work

## Abstract

**BACKGROUND:**

Management of rectal cancer has become more complex with multimodality therapy (neoadjuvant chemoradiotherapy and surgery) and this has led to the need to organize multidisciplinary teams. The aim of this study was to report on the planning, implementation and evaluation of an integrated care pathway for neoadjuvant treatment of middle and lower rectal cancer.

**DESIGN AND SETTING:**

This was a cross-sectional post-implementation study that was carried out at a public university cancer center.

**METHODS:**

The Framework for Program Evaluation in Public Health of the Centers for Disease Control and Prevention (CDC) was used to identify resources and activities; link results from activities and outcomes with expected goals; and originate indicators and outcome measurements.

**RESULTS:**

The logic model identified four activities: stakeholders’ engagement, clinical pathway development, information technology improvements and training programs; and three categories of outcomes: access to care, effectiveness and organizational outcomes. The measurements involved 218 patients, among whom 66.3% had their first consultation within 15 days after admission; 75.2% underwent surgery < 14 weeks after the end of neoadjuvant treatment and 72.7% completed the treatment in < 189 days. There was 100% adherence to the protocol for the regimen of 5-fluorouracil and leucovorin.

**CONCLUSIONS:**

The logic model was useful for evaluating the implementation of the integrated care pathways and for identifying measurements to be made in future outcome studies.

## INTRODUCTION

Colorectal cancer is the third leading type of cancer worldwide, accounting for about 1,200,000 new cases and 600,000 deaths annually.^1^ According to the National Cancer Institute of Brazil,^2^ approximately 34,280 new cases of colorectal cancer were expected to occur in this country in 2016.

About 25% of occurrences of colorectal cancer are located in the rectum. Over the last few decades, there have been major achievements in rectal cancer treatments, with the introduction of neoadjuvant therapy and total mesorectal excision for surgical removal of the tumor. Today, the treatment for middle and lower rectal cancer consists of three phases: first, the staging phase based on colonoscopy, computed tomography (CT) and magnetic resonance imaging (MRI) scans; followed by a second phase of neoadjuvant therapy with concomitant chemotherapy and radiotherapy (nCRT). The last phase is the surgery, including total mesorectal excision.

Integrated care pathways (ICPs) have been adopted into oncology practice as a tool for enhancing both quality and value by limiting undesirable variability and reducing cost, while providing the optimal course of care for a patient’s specific diagnosis.^3^ ICPs are structured multidisciplinary care plans that detail essential steps in the care for patients with a specific clinical problem. They support translation of clinical guidelines into local protocols and their subsequent application to clinical practice.^4^ ICPs have been implemented worldwide, but the reporting of the implementation processes is frequently poor and there is a lack of evidence about their impact.

**Figure 1.**
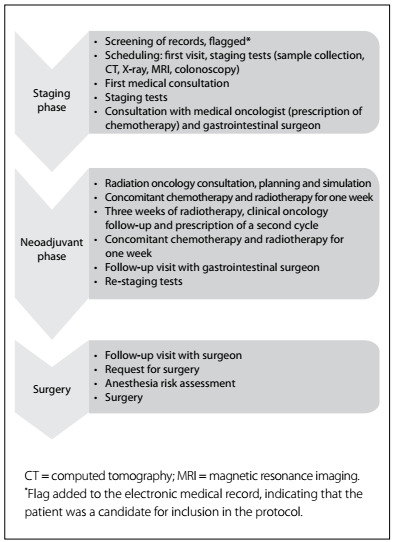
Flowchart of the integrated care pathway for rectal cancer that was started at the Instituto do Câncer do Estado de São Paulo, São Paulo, Brazil, in 2011.

In the present study, an ICP for neoadjuvant treatment of middle and lower rectal cancer was implemented at a public university cancer center with about 10,000 new cancer patients per year, across the state of São Paulo, Brazil. The planning and implantation of the ICP involved participation by medical oncologists, gastrointestinal surgeons, radiation oncologists, endoscopists, radiologists, pathologists, anesthesiologists, physicians, nurses, nutritionists, social workers, psychologists and physiotherapists. This multidisciplinary team standardized practices and constructed a flowchart outlining the sequence and timing of consultations, staging procedures, nCRT and surgery (Figure 1).

In order to report on the experience of implementing this ICP, a program logic model was used to inform the planning and development of the evaluation process. Logic models are defined as pictures of the way in which planners think their program is going to work. They comprise the theory and assumptions underlying the program.^5^

**Figure 2.**
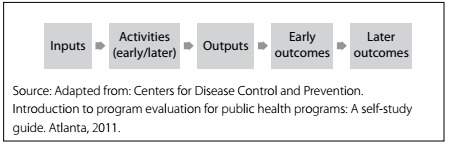
Logic model.

Logic models originate from the field of program evaluation and are diagrams that convey relationships between contextual factors, inputs, processes, program activities and intended outcomes.^6^^,^^7^^,^^8^ They may depict all or some of the following basic components: inputs, activities, outputs and outcomes (Figure 2). Inputs refer to the resources that go into the program, for it to perform its planned activities, and these can include human, financial, organizational and community resources. Activities refer to processes, tools, events, technology and actions that are implemented through the program and by its staff, in relation to the target population. Outputs are the direct products of program activities, usually measured in countable terms (e.g. the number of multidisciplinary meetings held or the number of first medical consultations booked). Outcomes are the changes that result from the activities and outputs of the program. They describe specific changes to the behavior, knowledge, skills, status and level of functioning of the target population for the program. In summary, logic models are flowcharts that display a logical sequence of steps in program implementation and achievement of desired outcomes.^8^

They have been used in a variety of fields,^9^ and there is growing recognition of their importance in the planning, implementation and evaluation of funded programs. For example, the United States Centers for Disease Control and Prevention (CDC) have used logic models^8^ to evaluate the effectiveness of public healthcare programs and show the success of these programs in achieving intended outcomes, to key stakeholders.

As far as we know, no studies on ICPs for neoadjuvant treatment of middle and lower rectal cancer, with analysis using program logic modelling, had previously been conducted. Furthermore, standardization of treatment for this type of cancer at our institution was not an easy task: there had been complaints about delays in radiotherapy, examinations and surgery; time interval measurements between the phases of treatment were unknown; and there were difficulties in managing all the steps of the forms of rectal cancer care that were in use.

## OBJECTIVE

The aim of this study was to report on the planning, implementation and evaluation of an ICP for rectal cancer treatment, using a logic model.

## METHODS

This is a cross-sectional post-implementation study reporting the implementation of an administrative and healthcare program of cancer care at the Cancer Institute of the State of São Paulo (Instituto do Câncer do Estado de São Paulo, ICESP), São Paulo, Brazil, 2011-2013. A program logic model approach was adopted with the aim of designing an evaluation that would focus on relevant healthcare outcomes (access to care, effectiveness of care and organizational outcomes) and factors that were involved in achieving these outcomes, using the CDC’s Framework for Program Evaluation in Public Health.^8^ Thus, a set of flow charts that displayed the sequence of logical steps and desired outcomes was used to link the key elements of the model: inputs, activities, outputs, early outcomes and later outcomes.

In the present study, the development of the logic model began with a review of the literature. This identified thinking, policy and research relating to colorectal cancer treatment and the role of ICPs in the delivery of care, both in Brazil and in other countries. It also involved a review of policy and program documents and one-to-one interviews with a sample of six managers and thirteen healthcare professionals involved in the development and delivery of the ICP. This phase resulted in identification of program goals, objectives and inputs.

The inputs were listed as the service users (patients included in the care pathway) and the resources (human resources and facilities) that were needed to carry out activities (Table 1).

**Table 1.** Logic model - Inputs and activities

Background	Period prior to implementation of the integrated care pathway: A public teaching hospital specializing in oncology opened its doors in May 2008, to treat public healthcare system patients who had been diagnosed with cancer. Patients were admitted by medical oncologists or surgeons. Although the established multimodal treatment for middle or lower rectal cancer consisted of neoadjuvant chemoradiotherapy followed by surgical resection, there was no coordination between the phases, which harmed the continuity of care. Until 2010, radiotherapy was done in a different service.
Goal	To implement an integrated care pathway for neoadjuvant treatment of rectal cancer, consisting of radiotherapy with 5040 cGy delivered in 28 fractions (540 cGy in the boost phase and 4500 cGy in the pelvic phase), over a five-week period. Concomitant chemotherapy (FULV regimen^12^ with 350 mg/m^2^ of 5-fluorouracil and 20 mg/m^2^ of leucovorin) was delivered as two five-day courses during the first and fifth weeks of radiotherapy. Surgery with total mesorectal excision consisted of open rectosigmoid resection (ORR), laparoscopic rectosigmoid resection (LRR) or abdominoperineal resection (APR).
Objectives	To manage all steps of the treatment for middle and lower rectal cancer and provide multidisciplinary continuity of care.
Inputs	
1. Service users	Inclusion Criteria: Patients with rectal cancer Exclusion Criteria: Patients with metastatic disease at diagnosis Patients who were unable to undergo neoadjuvant treatment: clinical condition precluded the use of nCRT; or immediate surgery was indicated; or a rapid course of neoadjuvant radiotherapy was indicated Patients who had previously been treated for cancer Patients who had not adhered to the nCRT regimen
2. Resources	Human Resources: medical oncologists, gastrointestinal surgeons, radiation oncologists, endoscopists, radiologists, pathologists, anesthesiologists, physicians, nurses, dieticians, social workers, psychologists, physiotherapists, hospital administrators and data managers
	Facilities: chemotherapy sector, radiotherapy sector, operating rooms, inpatient units, consultation rooms, imaging service and electronic medical records
Activities	
1. Stakeholder engagement	Clinical staff engagement: Multidisciplinary meetings were held under the leadership of a board of directors. Medical oncologists, surgeons, radiation oncologists, radiologists, pathologists, clinicians and anesthesiologists reviewed the neoadjuvant treatment protocol for middle and lower rectal cancer and defined the intervals between the phases of the treatment.
2. Clinical pathway development	An integrated care pathway was designed as a flowchart by the administrative group. Identification of patients’ input into the clinical pathway Definition of the time interval between record screening and the first medical consultation Booking first medical consultations on the pathway Staging test standardization Definition of term reports Sharing of chemotherapy and radiotherapy session schedules Active monitoring of surgery requests Definition of time interval between neoadjuvant treatment and surgery
3. Information technology improvements	Enablement of pathway patient identification using a flag added to the electronic patient charts Development of a report to identify pathway patients who have consultations and tests scheduled Development of a report to identify pathway patients who do not have any scheduling Development of a report to calculate dates of future steps on the pathway, to help in reception sector scheduling Development of the flag deactivation process
4. Training program	Training program for outpatient reception workers to enable schedule tests and consultations in accordance with the flowchart Training program to enable use of the reports that have been developed Training program to activate flags: regulation sector Training program to deactivate flags: physicians

The next phase involved identification of all the activities (services or interventions) that were developed as requirements for fulfilling the implementation goals. The direct results from the activities (outputs) were linked to expected goals, which originated indicators and measurements for evaluating pathway outcomes (Table 2). The results from the activities (outputs) were evaluated in accordance with the goals and were expressed as the percentage of patients who achieved the goal.

**Table 2.** Pathway implementation - outputs, outcomes and measurements/indicators

Concept and evaluation criteria	Measurements/indicators	Goal	Description	Source
Outputs				
1. Pathway implantation	Time interval between electronic medical record flagging and first consultation (days)	≤ 15 days	mean, % patients within the target	electronic health records
	Time interval between first medical consultation and start of neoadjuvant treatment (days)	≤ 46 days	mean, % patients within the target	electronic health records, radiation therapy system
	Time interval between start and end of neoadjuvant treatment (days)	≤ 45 days	mean, % patients within the target	radiation therapy system
	Time interval between end of neoadjuvant treatment and surgery (weeks)	≤ 14 weeks	mean, % patients within the target	electronic health records
	Time interval between first medical consultation and surgery (days)	≤ 189 days	mean, % patients within the target	electronic health records
	Adherence to FULV regimen protocol for concomitant chemotherapy	100%	% adherence	electronic health records
Early outcomes				
1. Access to care	Time interval between admission and first consultation (days)	Comparison of the measurements of pathway implementation with the period before ICP implementation	mean, % patients within the target	electronic health records
	Time interval between first medical consultation and start of neoadjuvant treatment (days)	Comparison of the measurements of pathway implementation with the period before ICP implementation	mean, % patients within the target	electronic health records
	Time interval between end of neoadjuvant treatment and surgery (weeks)	Comparison of the measurements of pathway implementation with the period before ICP implementation	mean, % patients within the target	electronic health records
2. Effectiveness of care	Time interval between first medical consultation and surgery (days)	Comparison of the measurements of pathway implementation with the period before ICP implementation	mean, % patients within the target	electronic health records
3. Organizational outcomes	Resource use	Comparison of the measurements of pathway implementation with the period before ICP implementation	numbers of consultations, CT scans, MRI scans, colonoscopies and radiotherapy sessions	administrative database
Later outcomes				
1. Effectiveness of care	Overall survival time	Comparison of the measurements of pathway implementation with the period before ICP implementation	survival curves	retrospective cohort study
2. Organizational outcomes	Cost evaluation	Comparison of the measurements of pathway implementation with the period before ICP implementation	cost of treatment	retrospective cost analysis

FULV: 350 mg/m^2^ of 5-fluorouracil and 20 mg/m^2^ of leucovorin, which was used as the regimen protocol for concomitant chemotherapy.

CT = computed tomography; MRI = magnetic resonance imaging; ICP = integrated care pathway.

All consecutive patients with middle or lower rectal cancer who were admitted to the public university cancer center between May 2011 and December 2013 were evaluated. These patients were named the ICP group (ICPg). Patients who had undergone prior treatment, those who had not undergone nCRT treatment and those who presented metastatic disease at diagnosis were excluded.

A single database in Microsoft Excel was built up, using information extracted from the following electronic healthcare records: Tasy system (Philips Clinical Informatics, Blumenau, Brazil), Laserfiche document scanning system (Long Beach, CA, USA), Mosaiq radiation therapy system (Elekta AB, Stockholm, Sweden) and the hospital cancer registry (HCR). The following information was obtained: the date when the patient was included in the ICPg, the date of the first medical consultation, the start and end dates of nCRT and the date of the surgery.

Statistical analyses were performed using the Statistical Package for the Social Sciences, version 18.0 for Windows (SPSS Inc., Chicago, IL, USA). Qualitative variables were shown as counts and percentages. Means, medians, standard deviations and 95% confidence intervals (95% CI) were calculated for quantitative variables. The significance level adopted for all statistical tests was 5%.

This study was approved by the Research Ethics Committee of the University of São Paulo (São Paulo, Brazil), under protocol no. 126/14, on May 9, 2014.

## RESULTS

The clinical staff and the administrative team were the stakeholders in developing the ICP. They were the people with an interest in the results from the evaluation and were the intended users of its findings. A board of medical oncologists, surgeons, radiation oncologists, radiologists, pathologists, clinicians and anesthesiologists was assembled with the aim of reviewing the neoadjuvant treatment protocol for middle and lower rectal cancer. Multidisciplinary meetings were held under the leadership of the board to discuss treatment steps and intervals between phases.

The clinical protocol consisted of radiotherapy, with 5,040 cGy delivered in 28 fractions (540 cGy in the boost phase and 4,500 cGy in the pelvic phase), over a five-week period. Concomitant chemotherapy (FULV regimen,^10^ comprising 350 mg/m^2^ of 5-fluorouracil and 20 mg/m^2^ of leucovorin, intravenously, on days D1-D5) was delivered in two courses during the first and fifth weeks of radiotherapy. The surgery, with total mesorectal excision, consisted of open resection (ORR), laparoscopic resection (LRR) or abdominoperineal resection (APR) of the rectum and sigmoid.

The team of medical oncologists, surgeons and regulators established a record screening system in order to include patients in the ICPg. In this, a stamp placed on the patient’s admission chart was used to signal and identify new patients for the regulation sector.

Based on the ideal 15-day interval for the first consultation that had been established by the medical team, the outpatient reception sector reserved vacancies within the medical schedules to guarantee slots for first consultations with the medical oncologist and gastrointestinal surgeon for ICPg patients.

The medical oncologists and surgeons defined the types and quantities of laboratory tests, imaging scans and colonoscopies for staging. In accordance with the opinions of radiologists, pathologists, endoscopists and reception staff, they defined a desirable range of 15 days between the tests and the follow-up medical consultation.

The radiotherapy and chemotherapy sectors, nurses and reception staff developed a shared spreadsheet for concomitant sessions of chemotherapy and radiotherapy. Their aim was to ensure mutual real-time viewing of both sectors in the first and fifth weeks of neoadjuvant treatment.

The surgery scheduling sector developed a worksheet to monitor ICPg patients. This contained the following information: start and end dates of the nCRT, expected date for surgery, expected date for clinical and anesthesia risk assessment, date of clinical and anesthesia risk assessment, expected date for surgical scheduling request and date of the surgery. The goal of the worksheet was to monitor and advise patients based on the expected dates, in order to schedule the procedure at a time close to the ideal for performing the surgery after nCRT.

Improvements to the Tasy electronic health record system needed to be developed by the information technology sector in order to accomplish the ICP. An alert in the patients’ medical chart, called a flag, was created to allow both the administrative and the care team to identify each ICPg patient. This flagging would appear on the initial screen for patient chart users, as the following information: “Integrated care pathway for neoadjuvant treatment of rectal cancer”. It was decided through reaching a consensus that the regulation sector would be responsible for inserting the flagging.

Several reports were developed within the Tasy electronic health record system. A report was created to make it possible to see which patients had been flagged but did not have any consultations scheduled. This was used by the administrative team to recall patients who were in this situation. Another report was developed to allow outpatient scheduling by the reception sector without the need for the flowchart steps and deadlines to be memorized. This report calculates the dates of future steps from the first medical consultation, according to the schedules available.

It was also necessary to develop a process to deactivate the flagging in the cases of patients who should not be in the pathway (due to metastatic disease, for example) or patients who had completed the pathway: doctors and/or the regulation sector staff could proceed with deactivation of the flagging.

A training program was developed in order to introduce the ICP step-by-step to the administrative sectors. Outpatient reception sectors were systematically trained to use the reports, with the aim of ensuring correct scheduling of medical consultations and tests.

The regulation sector staff was trained to flag eligible patients who had been identified by the medical teams through screening the documentation of patients who had been referred to the hospital.

The information technology sector developed a training manual for flag deactivation. This manual was presented at medical meetings and has been made available via e-mail to medical oncologists and surgeons.

Table 1 shows the inputs and activities of the logic model for ICP implementation.

Table 2 shows the outputs, outcomes and indicators selected for evaluating pathway implementation: five relating to time interval measurements between the phases of treatment and one relating to adherence to the FULV regimen protocol for concomitant chemotherapy. An indicator target was established by the multidisciplinary team during the development of the ICP. Measurements of access to care, effectiveness of care and organizational outcomes were selected for evaluating the early and late outcomes (Table 2).

Table 3 presents the results from the measurements and indicators relating to ICP implementation. A total of 413 patients who had been diagnosed with rectal cancer were admitted to the service between May 2011 and December 2013. Among these, 195 were excluded (92 whose clinical condition precluded the use of nCRT or who required immediate surgery; 21 who had previously been treated for cancer; 74 who presented metastatic disease at the time of the diagnosis; and eight who had not adhered to the nCRT regimen). Therefore, the measurements involved 218 patients, who were named the ICP group (ICPg): 66.3% had their first consultation within 15 days after admission; 67.9 started the nCRT within 46 days after their first consultation; 89.9% completed the nCRT regimen within 45 days; 75.2% underwent surgery within 14 weeks after the end of neoadjuvant treatment; and 72.7% completed the treatment within 189 days. The rate of adherence to the FULV regimen protocol was 100%.

**Table 3.** Measurements/indicators of pathway implementation among patients treated for rectal cancer at the Instituto do Câncer do Estado de São Paulo, São Paulo, Brazil, 2011-2013

Measures/indicators	Indicator goal	% ICP within goal	ICP (n = 218)
∆ EMR flagging - first consultation (days)			
mean (SD)	≤ 15 days	66.3	12.7 (8.8)
95% CI			(11.5-14.1)
median			13.0
∆ first consultation - start of nCRT (days)			
mean (SD)	≤ 46 days	67.9	48.4 (29.8)
95% CI			(44.3-52.3)
median			39.0
∆ first - last nCRT session (days)			
mean (SD)	≤ 45 days	89.9	40.1 (6.7)
95% CI			(39.2-41.0)
median			39.0
∆ last nCRT session - surgery (weeks)			
mean (SD)	≤ 14 weeks	75.2	14.8 (4.6)
95% CI			(14.2-15.4)
median			13.2
∆ first consultation - surgery (days)			
mean (SD)	≤ 189 days	72.7	192.0 (45.8)
95% CI			(185.9-198.1)
median			177.0
Adherence to FULV1 regimen protocol	100%	NA	100%

ICP = integrated care pathway: patients were admitted between May 12, 2011, and December 31, 2013 (after implementation of the ICP); EMR = electronic medical record; NA = not applicable; nCRT = neoadjuvant chemoradiotherapy; ∆ = time interval between; ^1^FULV = 350 mg/m^2^ of 5-fluorouracil and 20 mg/m^2^ of leucovorin.

## DISCUSSION

ICP is an administrative and care milestone that combines administrative support with care needs in order to ensure multidisciplinary care. Implementation of a clinical pathway within daily practice is challenging, especially in public hospitals with high demand and limited resources.

Regarding pathway implantation, the initial activity of engaging stakeholders showed that there was a need to standardize and disseminate the clinical pathway between the various medical specialties and find solutions to ensure that the treatment steps were achieved. Previously, referral to another team or to the next stage was done only after the end of the preceding stage. Some adaptations were made because of a lack of time resources: for example, the medical oncologists prescribed chemotherapy until chemoradiotherapy sessions started to be scheduled, because of difficulties in coordinating the sessions. Scheduling the surgery at the right time after neoadjuvant treatment was also a challenge.

In this regard, the National Comprehensive Cancer Network, through its clinical practice guidelines for oncology, advocates a multidisciplinary approach involving oncologists, gastroenterologists, surgeons, radiation oncologists and radiologists.^11^ Some institutions have organized their multidisciplinary teams through systematic meetings, in the form of “tumor boards”.^12^ However, there is a lack of research demonstrating the effectiveness of the multidisciplinary approach.^13^^,^^14^^,^^15^^,^^16^

To develop the clinical pathway, administrative support was necessary to ensure that the flowchart design defined by the medical teams within daily practice was implemented. The care teams (multiprofessional and medical) raised any critical issues and needs that had to be resolved.

Regarding inputs and activities, communication problems between the teams were a barrier that needed to be overcome. The gap between the care team and the administrative team is an aggravating factor: on one hand, the care team perceives the administrative team to be a bureaucratic control sector focused exclusively on productivity; on the other hand, the administrative team perceives the care professionals to be technical experts who excessively request supplementary tests and resources without having any management experience. Data in the literature have demonstrated that there is a need for evaluation studies on clinical pathways, in order to check the proposed interventions, behavioral changes and context, and to identify the critical success factors.^17^

Flagging (i.e. stamps that were placed on the regulatory documentation) and shared spreadsheets were simple solutions that were developed to enable communication between the stakeholders. Another critical point was the need to rationalize resources and processes. Previously, a diversity of diagnostic tests had been requested by doctors and there had been delays in issuing imaging examination reports. Through defining staging tests and interval deadlines, rational use of resources became possible.

The training program was especially necessary because of the high turnover rate in the outpatient reception sector. Several meetings were held during the implementation of the ICP: between medical teams, between medical and care teams and between care and administrative teams. These interactions brought the various professionals together and facilitated mutual understanding of their respective attributions, thus placing value on the importance of each professional within the strands of the clinical pathway.

Finally, regarding outputs and outcomes, it has been pointed out in the literature that there is a lack of indicator descriptions for colorectal cancer protocols. Ludt et al.^18^ developed a list of 52 quality indicators to cover relevant aspects of the treatment of colorectal cancer, among which 11 related to diagnostic procedures, 28 to therapeutic management, six to follow-up and seven to the patient’s perspective. These authors noted that there was some difficulty in putting the indicators into operation because of a lack of data source specification and collection methods. They also showed that indicators focusing on the surgical treatment predominated and pointed out that there was a need to measure the quality of care.

In this study, the indicators showed opportunities for improvement. Specific studies and actions are needed in order to increase the percentage of patients with ranges of values for these indicators that are within the targets.

Management of middle and lower rectal cancer has become complex with the multimodality therapy of nCRT and surgery. This has led to a need to monitor access to all phases of the treatment. Eldin et al. showed that there were difficulties in relation to adherence to treatment guidelines among stage II/III rectal cancer patients in Alberta, Canada, because of lack of access to medical oncologists among patients, and the distance from these patients’ homes.^19^ Gallego-Plazas et al. evaluated rectal cancer treatment in a tertiary-level hospital and pointed out that delays in the intervals between the different phases of treatment and lack of coordination were critical factors.^20^

In relation to effectiveness of care, there is no agreement regarding the impact on overall survival of multimodal treatment for rectal cancer. Wiegering et al. reported that increased use of neoadjuvant therapy and total mesorectal excision led to improvement of overall survival.^21^ Chang et al. also reported that use of neoadjuvant treatment was increasing but did not find any differences in five-year overall survival.^14^

The organizational outcome indicators selected in the present study were related to resource use and cost evaluation. Although use of integrated care pathways has been correlated with improvement to the quality of care, cost reduction and optimization of resource allocation,^22^ few studies have quantified their effectiveness.

Some limitations of the present study can be highlighted. Firstly, early and later outcomes (**Figure 4**) were not evaluated separately. Furthermore, since the purpose of the study was to analyze an ICP implementation process, outcomes before and after the intervention were not compared. Secondly, it might be argued that a wider group of participants could have been included to reflect differences in views among participants from similar backgrounds. Although the proposal to evaluate ICPs for rectal cancer treatment came from a hospital/university joint research network and members of this network formed the research team, inclusion of a wider group of stakeholders in the process generated further ownership and support for the subsequent evaluation. Thus, we believe that this exercise was conducted among a reasonably coherent group of stakeholders, across the range of roles involved in rectal cancer treatment.

We found that the logic model was an effective planning and evaluation tool and a useful project management resource that greatly increases the likelihood that ICP goals would be reached, consistently with these aims. However, some of the difficulties in developing a logic model were significant, including the availability of time among the stakeholders, the requirement for trained staff to conduct the evaluation process and the need for institutional commitment to the project.

Future studies should provide comparisons with the period before the implementation of the ICP, in order to evaluate early outcomes relating to access to care (reduction of the time intervals of the treatment), effectiveness of care (reduction of the total duration of the treatment) and organizational outcomes (resource use).

## CONCLUSIONS

Implementation of an ICP for rectal cancer treatment, analyzed by means of a logic model approach, was feasible and informed the design of this complex intervention for evaluation of rectal cancer care.
